# Kinetics of the radiative and nonradiative recombination in polar and semipolar InGaN quantum wells

**DOI:** 10.1038/s41598-020-58295-x

**Published:** 2020-01-27

**Authors:** Lucja Marona, Dario Schiavon, Michał Baranowski, Robert Kudrawiec, Iza Gorczyca, Anna Kafar, Piotr Perlin

**Affiliations:** 1 Institute of High Pressure Physics PAS, Sokolowska 29/37, 01-142 Warsaw, Poland; 2 TopGaN, Sokolowska 29/37, 01-142, Warsaw, Poland; 3 Departament of Experimental Physics, Wroclaw University of Science and Technology, Wybrzeże Wyspiańskiego 27, 50-370 Wrocław, Poland

**Keywords:** Diode lasers, Fluorescence spectroscopy

## Abstract

We studied mechanisms of recombination in InGaN quantum wells in polar and semipolar structures. Photoluminescence measurements show that the optical emission linewidths for polar and semipolar structures are almost identical suggesting the same level of indium fluctuations in quanutm wells. Their “peak-energy-versus-temperature” relations demonstrate very pronounced “s-shape” effect. Emission linewidth measured by cathodoluminescence does not depend on area from which the light is collected meaning that the fluctuations are smaller that 100 nm. The time scale of recombination process are of the order of 80 ns for polar and 2 ns for semipolar. Energy dispersion of the recombination time is strong in polar structures and very weak in semipolar ones which can be interperted in terms of electric field influence on photoluminescence lifetime energy dispersion. At room temparture emmission is dominated by Schockley-Hall-Read recombination and does not show any dispersion. Rate equation analysis of photoluminescence transients show domination of excitonic recombination in the case of polar samples (low temperature) and bimolecular in the case of semipolar ones. Both types of quantum wells, polar and semipolar look similar from the point of view of localization but differ in their radiative recombination mechanisms.

## Introduction

III-nitrides based laser diodes and light emitting diodes are presently an indispensable part of the modern optoelectronics. Nitride based devices are widely used as sources of coherent and incoherent light from UV^1^ to the green part of spectrum^2^. They also serve as a very efficient and economical replacement of Edison’s bulb^3^. From the physical perspective, nitride semiconductors are the first wide band gap materials of non-cubic structure used for the mass production in electronics and optoelectronics. This means that we encounter a range of new phenomena not observed previously in conventional III-V devices, like gallium arsenide. For visible optoelectronics, a crucial element of nitride emitters is an InGaN quantum structure. The InGaN quantum wells (QWs) are strongly strained due to 10.6% of lattice mismatch between InN and GaN^3^. The strain in the case of wurtzite structure at standard polar (0001) orientation of the growth surface, leads to the appearance of several MV/cm range electric field across the quantum system. The large internal electric field manifests through the Quantum Confined Stark Effect (QCSE)^4^, which consists in the spatial separation of the electron and hole wave functions, thus the reduction of the overlap of their wavefunctions^5^ and consequently reduction of the recombination efficiency. The radiative lifetime increases by 1–2 orders of magnitude exposing the carriers to much stronger competition from nonradiative processes.

The other important aspect of InGaN QWs physics is a susceptibility of this compound to the composition fluctuations, a process initiated by the presence of large internal strain, connected with the difference in Ga-N and In-N atomic bond lengths and very different thermodynamic stability of InN and GaN binary components. Localization of carriers by In-composition fluctuations was claimed to be a source of the success of nitride based light emitters. Chichibu’s paper^5^ suggested that the In-composition fluctuations in InGaN layers enhance the radiative recombination efficiency, by suppressing the carrier diffusion into nonradiative recombination centers. However, with the development of nitride laser diodes, the beneficial role of the fluctuations of In-composition has been frequently questioned.

While the presence of the internal electric field and indium fluctuations, even if not beneficial, seems not to be detrimental for devices like light emitting diodes (LEDs), both of them are harmful for laser diodes. The composition fluctuations lead to spontaneous emission broadening, gain dilution and higher optical absorption losses. Internal electric fields via QCSE cause the optical gain reduction. The obvious way of eliminating QCSE is to grow the structure along a nonpolar or semipolar direction of the wurtzite crystal, what dramatically reduces the internal electric fields. A good quality laser diodes grown on different than *c*, GaN planes have been demonstrated during the last decade^2,6,7–11^.

Within this work, by comparing the emission spectra of polar and semipolar InGaN structures and their behavior with temperature and time, we present new evidences on the mechanisms of recombination in InGaN QWs. By choosing substrate polarity we modify the internal electric field in the investigated structures. To look more deeply into the relations between radiative and non-radiative recombination in differently oriented InGaN QWs we use time resolved photoluminescence (PL). To better control the influence of the fluctuations of composition we probe small areas of the InGaN structures by using highly spatially resolved cathodoluminescence (CL).

To perform experimental analysis we use polar *c-*plane (*P*) and semipolar (20–21) plane (*S*) sets of samples. The samples were fabricated in the same growth run by Metalorganic Vapor Phase Epitaxy (MOVPE).

**Figure 1:**
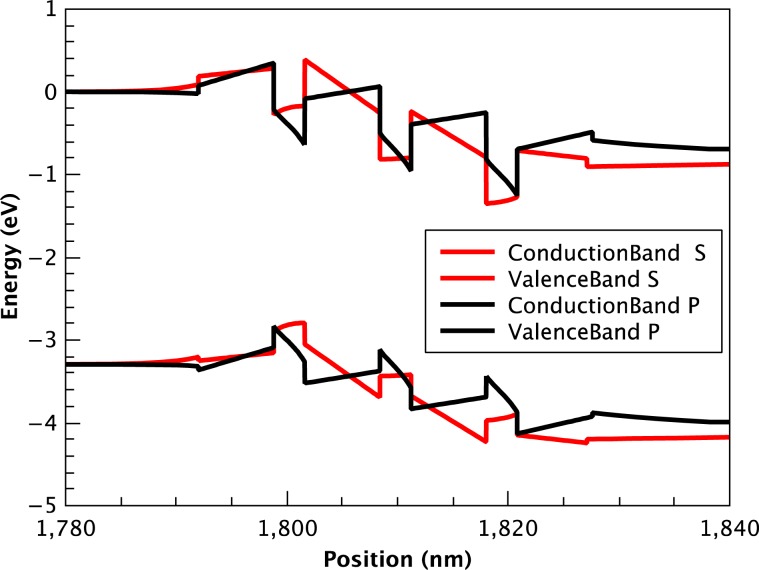
Calculated band profiles for structures *P* and *S* at applied voltage 4 V.

To illustrate the influence of the internal electric field on the band edge profiles in polar (*P*) and semipolar (*S*) structures, the calculated conduction and valence bands along the growth direction of In_x_Ga_1−x_N/GaN quantum structures with *x* = 0.22, corresponding to the In-content in our samples, are presented on Fig. 1. The calculations were performed using ‘Silense’ package.

## Results

### Emission spectra of polar and semipolar InGaN QW structures

**Figure 2:**
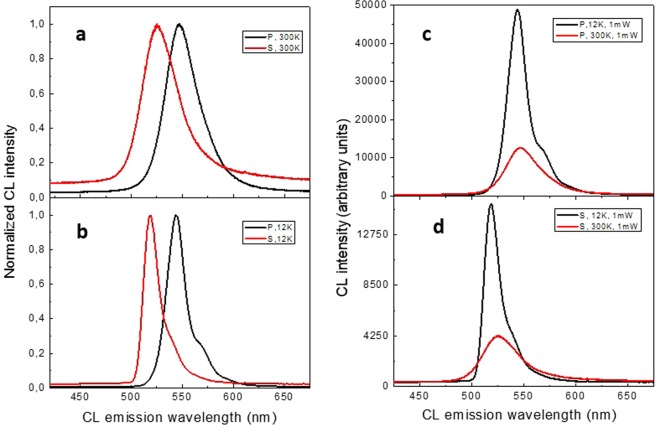
PL spectra for samples: (**a**) *P* and *S* at T = 300 K, (**b**), *P* and *S* at T = 12 K (**c**) *P* at T = 12 K and T = 300 K, (**d**) *S* at T = 12 K and T = 300 K. Spectra measured at low temperature show characteristic LO phonon replica.

Figure 2 shows PL spectra for two chosen samples of In_0.3_Ga_0.7_N/GaN structures – polar (*P*) and semipolar (*S*) with identical QW composition and thickness of 2.3 ± 0.15 nm, determined by x-ray diffraction. Measurements were performed at low temperature (LT), T = 12 K, and at room temperature (RT), T = 300 K. One can easily see (Fig. 2a) that in LT regime the position of the emission peak, which in the case of *S* sample is 520 nm, is shifted in the sample *P* towards longer wavelengths (550 nm). Similar effect is observed at RT (Fig. 2b). The difference in the peak positions can be explained by QCSE, which causes the ‘red shift’ of energy in the polar sample *P*. In both samples we observe almost the same shift of the energy peaks with temperature and analogical band broadening, also the change of the peak intensity with temperature is similar for both samples, as it is illustrated in Fig. 2c,d.

Observed at LT weak peak, about 90 meV below the main peak, is its phonon replica. The resolution of the phonon replica from PL spectum is possible due to a good quality of the samples which is manifested by the weak broadening of PL spectra. Interestingly, the full width at half maximum (FWHM) of the emission peaks are almost the same for both samples - at RT it is 39 nm for *P* and 40 nm for *S* sample (Fig. 2b). The linewidth of the luminescence should be the reflection of the system disorder, and the natural way of thinking is to associate this disorder with local variation of In composition and/or QW thickness. However, the problem of the emission linewidth in InGaN quantum structures has been discussed over last two decades. In the early work of O’Donnel *et al*.^12^, the remarkable, universal character of the emission broadening in InGaN QWs and epilayers was reported. Authors argued that since the line broadening is apparently independent of the growth method, it must reflect an inherent feature of these structures. To our knowledge there is no up to date any good model directly linking the emission linewidth with other observable properties of InGaN structures.

As the emission linewidth is commonly associated with the inhomogeneus broadening, and consequently with the structural disorder, we decided to look more closely on the observable fluctuation scale using cathodoluminescence (CL) mapping. Let’s imagine monochromatic cathodoluminescence image reflecting indium inhomogenities in quantum wells. If we collect the light emitted from the whole analyzed area of non uniform structure we should expect broad emission spectra. In contrast, if we gather the signal from region of the size smaller that the characteristic radius of the indium fluctuations, emission spectra suppose to be much narrower.

**Figure 3:**
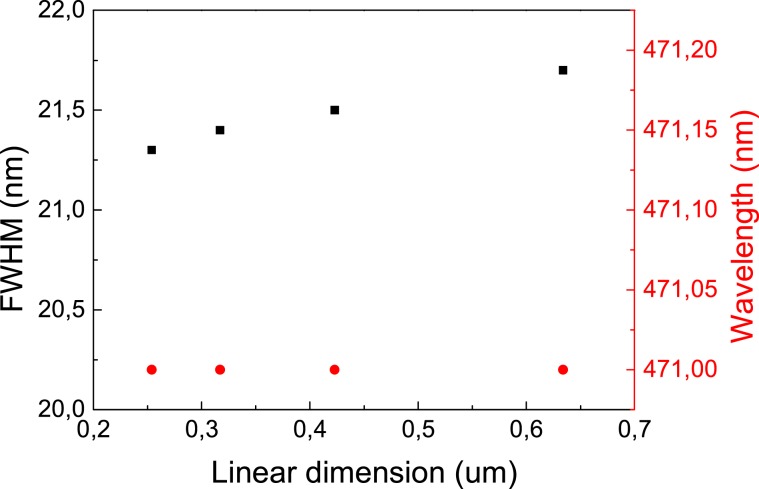
The emission linewidh versus the CL signal collection region diameter for sample P.

We collected the monochromatic cathodoluminescence image at the quantum wells emission energy. To excite QWs emission we applied 15 kV acceleration voltage. Then, we measured emission spectra gathering signal from the large area. Subsequently, we were changing the magnification of the microscope until we observed an uniform image. The FWHM of the spectra collected at various magnications of the microscope are presented on Fig. 3.

We can observe in Fig. 3 that over large span of image diameter, the linewidth remains almost constant, meaning that the visible in CL In fluctuation landscape does not practically influence on FWHM. Most likely the observed source of broadening is related to submicron size inhomogeneities causing strong localization.

### “S-shape” behavior of the emission peak positions

One of the very characteristic optical effects observed in InGaN ternary alloys (predominantly in QWs structures) is nonmonotonic, „s-shaped“ change of the emission peak position with the temperature^13–15^. An observation of the “s-shape” behavior of the emission energy would be considered as a manifestation of carrier localization and it has been a common way of explaining and grading the degree of fluctuations present in InGaN structures. Therefore, measurement of the emission peak position as a function of temperature for *P* and *S* samples was an obvious next step in our study.

**Figure 4:**
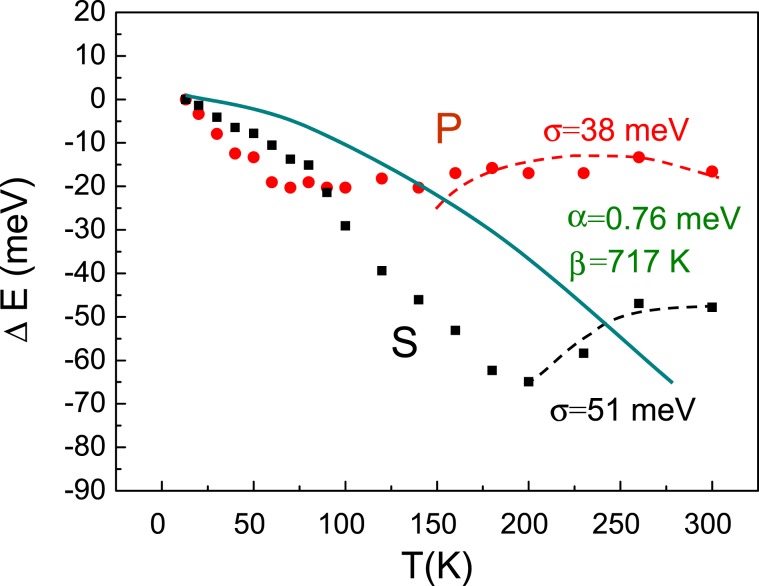
Temperature dependence of PL peak position energy for *P* and *S* sample. Solid lines represent Varshni formula for avarege content of In_30_Ga_70_N^13^ and dashed lines Eliseev model fitted to the experimental data^17^.

In Fig. 4 the temperature dependence of PL peak position energy for *P* and *S* sample is presented. “s-shape” behavior of the peak position is clearly seen for both samples. For quite a long time the most adopted interpretation of the “s-shape” effect^14^ has been based on the thermal population of a complex landscape of potential, characteristic for a fluctuating ternary InGaN alloy. Accordingly, the first, low temperature, region corresponds to the increase of temperature to the level enabling the carrier’s relocation to the global potential minima (carrier thermalization). The second, medium temperature region, corresponds to thermal population of the density of states. Finally, the third, high temperature region, is a normal energy gap shrinkage commonly described by the Varshni function^13^ (temperature up to RT). Both samples are characterized by a very pronounced I region, visible II region and weakly formed region III of the “s-shape”. In the *S* sample the onset of region II is shifted towards higher temperatures, up to almost 200 K. Within the classical interpretation that would mean that gaining the thermal equilibrium requires temperatures closer to the room temperature. Interestingly, in semipolar sample, the band gap (E_g_) reduction is not visible up to RT. As it can be deduced from Fig. 3, the total change of the emission energy through the temperature range 10–300 K is 50 meV for semipolar QW, and 18 meV only, for polar QW. This significant difference can originate from the trade off between temperature induced bandagap shrinkage and temperature induced screening of QCSE due to thermal activation of carriers. In general this phenomenon can be pronounced for polar QWs and weak for semipolar QWs.

Classically, in binary compounds, with uniform atom arrangment, the band gap energy dependence on temperature is described by Varshni^16^ formula:

1 $${{\rm{E}}}_{{\rm{g}}}({\rm{T}})={{\rm{E}}}_{{\rm{g}}}(0)-\,\frac{{{\rm{\alpha }}{\rm{T}}}^{2}}{{\rm{T}}+{\rm{\beta }}}$$

where E_g_(T) is the band gap at given temperature T, and *a,b* are fitting parameters. According to this formula, in InGaN alloy with average In content of 30% band gap changes by 70 meV between 0 and 300 K (a = 76 meV/K, b = 717 K^13^). However, this simple formula cannot be applied to the “s-shape” behavior of InGaN layers. Eliseev *et al*.^17^ proposed to add a new element to Varshni relation, which could descibe this behavior better:

2 $${{\rm{E}}}_{{\rm{g}}}({\rm{T}})={{\rm{E}}}_{{\rm{g}}}(0)-\frac{{{\rm{\alpha }}{\rm{T}}}^{2}}{{\rm{T}}+{\rm{\beta }}}-\frac{{\sigma }^{2}}{{k}_{B}T}$$

where σ is the width of Gaussian distribution of the density of states (density of states tail) and k_B_ is Boltzman constant. Presently this density of states tail is associted with deep localized states and reflects the degree of localization. This formula is able to describe region II and region III of the “s-shape”, however is unable to describe region I of the “s-shape”, because it assumes Boltzman statistics, inapplicable at low temperatures.

Varshni formula and Eliseev approach was fitted to our experimantal results, see Fig. 4. Varshni fitting was done taking into account emission energy at lowest temperature. Fitting does not match to experimental data as expected. Eliseev formula fits quite well to the blue shift region of the dependence. Our fitting parameters are *s* = *51 *meV for *S* sample, and s = 38 meV for *P* sample with the same a (0,76 meV/K) and b (717 K) as used in formula 1.

A different type of approach to “s-shape” is given by Langer *et al*.^18^. The authors propose to consider “blue” shift of the emission peak as the result of the balance of radiative and nonradiative recombination times in the presence of internal electric field. In brief, this model assumes that the broadening of the emission peak in InGaN QWs is related to the emission from the spatial regions characterized by different QW width and/or different QW composition (composition fluctuations understood within a segmented QW picture). Both effects influence, via QCSE, the wave function overlap and radiative recombination time. Langer proposes^18^ that the increase of the temperature causes shortening of the noradiative recombination time. This effect is supposed to kill the radiative recombination mostly in the regions where QCSE is large (long wavelength emission), because there, the radiative time is the longest, and accordingly, least competitive. Thus, the shortening of the nonradiative time leads to the elimination of the long wavelength part of the emission spectrum, producing an effective “blue” temperature shift. As both Eliseev^17^ and Langer^18^ models address only the *blue-shift* part of the s-shaped relation, applicability of these models is difficult to be judged from the temperature shift of the peak emission, only.

### Photoluminescence decay time

PL decay time measurement can bring additional insight to the nature of the radiative recombination. This is the experimental tool, which can be used to choose between the proposed recombination models, since the PL decay time is very sensitive to the magnitude of QCSE.

**Figure 5:**
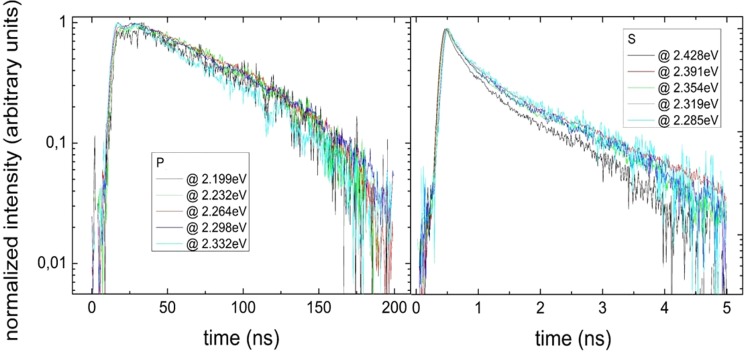
PL decay times measured at 12 K for polar (P), and semipolar (S) samples for different emission energies.

Figure 5 presents decay times for *P* (left) and for *S* (right) samples for different energies of the spectra. In the case of *S* samples the decay time is much shorter, as expected, due to larger overlap between wave functions of electrons and holes. This is an agrement with the previous reports^19^. Decay time at LT, corresponding to the radiative part of recombination, is equal to 87 ns for *P* sample, and only 2,4 ns for *S* sample. LT decay times for *P* and *S* samples show different nature; for *P* sample the dependence of PL intensity on time is almost exponential, whereas, for *S* sample nonexponential.

In order to better understand the physical nature of the recombination process we applied ABC^20^ rate equation to quantitatevely descripe photoluminescence transients. The standard formulation of ABC model is given by the equation:

3 $$\frac{{\rm{dN}}}{{\rm{dt}}}={\rm{An}}+{{\rm{Bn}}}^{2}+{{\rm{Cn}}}^{3}$$

where *n* is the carrier density, *A* = *A*_*ex*_ + *A*_*SHR*_, with coefficient *A*_*ex*_ corresponding to excitonic recombination, and *A*_*SHR*_ – to SHR nonradiative recombination, *B* is bimolecular recombination coefficient, and C is Auger recombination coefficient. Samples were excited by 400 nm frequency-doubled pulses (150 fs at 4.25 MHz) coming from a Ti: sapphire laser. The average power of exciting beam was 1 mW. Photogenerated carrier concentration was estimated to be 2.2 e 10^18^, (very short pulses, cannot provide more carriers). Auger recombination is not taken into account because of relatively low pumping densities used in this experiment, so we can neglect the coefficient *C*. Shockley Hall Read (SHR) recombination is usually neglected at low temperatures beacause of low population of phonons and though this approach is sometimes questioned we used as an acceptable aproximation, and thus *A*_*SHR*_(LT) = 0. The problem of nonradiative recombination in semiconductors is a complex effect of nontrivial description. The problem how to transfer the energy gap to many phonons instead of one photon was first considered in full extent by Lang *et al*. in 1977^21^. Both phonon cascade and multiphonon emission (MPE) were considered in their work. This was followed much later by the paper of Shenk^22^. They proposed that at low temperature the nonradiative recombination (SHR) lifetime should scale as (1/T)^3/2^, meaning that it is almost negligible at very low (cryogenic) temperature. This temperature relation is frequently used up to date. For instance, Okamoto *et al*.^23^ suggested that internal quantum efficiency of InGaN quantum wells reached 100% at 10 K. Similarly, the authors from other group experimentally determined nonradiative lifetimes in InGaN quantum wells, always showing strong increase at low temperature^24–27^. Although we are aware, that there is a possibility of the existence of a certain nonradiative recombination at very low temperature by for example multiphonon emission. In the present work we follow the common assumption that the nonradiative lifetime is long enough in comparison with the radiative one, making the radiative process dominating. Our approach is also based on the fact that all the samples studied were grown on low dislocation density samples of a good crystallographic quality. Transmission Electron Microscopy showed sharp interfaces and good homogeneity of the samples. Also the semipolar samples lacked the photoluminescence related to stacking faults, which frequently act as nonradiative recombination centers.

**Figure 6:**
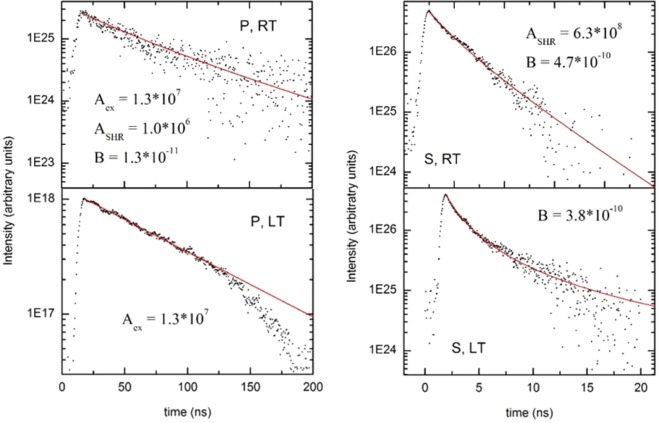
Decay times for *P* (**a**) and *S* sample (**b**) at LT and RT. Black dots correspond to experimental results, red curves represent fitting to ABC model (Eq. 3). The fitted values of A_SHR,_ A_ex_, and B coefficients are given for each case.

By fitting Eq. 3 to our experimental results for LT and RT we determine *A* and *B* coefficients. The results are presented in Fig. 6, and gathered for clarity in Table 1.

**Table 1:** Radiative (A_exc)_, nonradiative (A_SHR_) and bimolecular (B), recombination coeffcients, for polar (P) and semipolar (S) samples, fitted to experimental data measured at RT and at LT (12 K).

	A_exciton_ [s^−1^]	A_SHR_ [s^−1^]	B [cm^3^s^−1^]
P_RT_	1.3*10^7^	1*10^6^	1.3*10^−11^
P_LT_	1.3*10^7^	0	0
S_RT_	0	6.3*10^8^	4.7*10^−10^
S_LT_	0	0	3.8*10^−10^

Surprisingly, the fitting procedure reveal that at low temperature recombination in *S* sample is govered by bimolecular process (B) and at room temperature both, nonradiative SHR and bimolecular are important for the recombination. In contrary, in *P* sample, the PL decay time at LT is described by A_exc_ coefficient, only, and by all three recombination mechanisms (A_exc_, A_SHR_, B) at RT. According to common knowledge we should observe opposite situation – exctitons should be more pronunced in sample with smaller electric field, i.e., in *S* sample. Looking for an explanation of this phenomena we can imagine that all the mechanisms, nonradiative, bimolecular, and excitonic recombination, depend not exactly in the same manner on the electric field. In *P* sample, at LT, excitons can be frozen at local potential minima due to the composition fluctuations. If we imagine that in *S* sample fluctuations are smaller – then excitons are almost free and they are not so stable as in the case of polar QWs.

### Energy dispersion of the recombination time

If the observed light emission originates from different regions of an inhomogeneous material or/and have complex character being a mixture of emissions occurring via various physical mechanisms, then we can expect that the components of radiation may have various recombination times. This is a base of so called energy-dispersion of recombination time analysis. It has been observed in the past by many groups, that the recombination lifetime for InGaN QWs luminescence depends strongly on the emission energy, i.e. recombination time increases when we move towards lower energy side of the peak^3^. This observation was interpreted as a manifestation of high carrier localization. However, Langer *et al*.^19^ observed that in nonpolar InGaN QWs there is no any substantial dispersion of the PL lifetime. This observation was explained by associating the energy dispersion of the radiative recombination time with the presence of the internal electric field.

**Figure 7:**
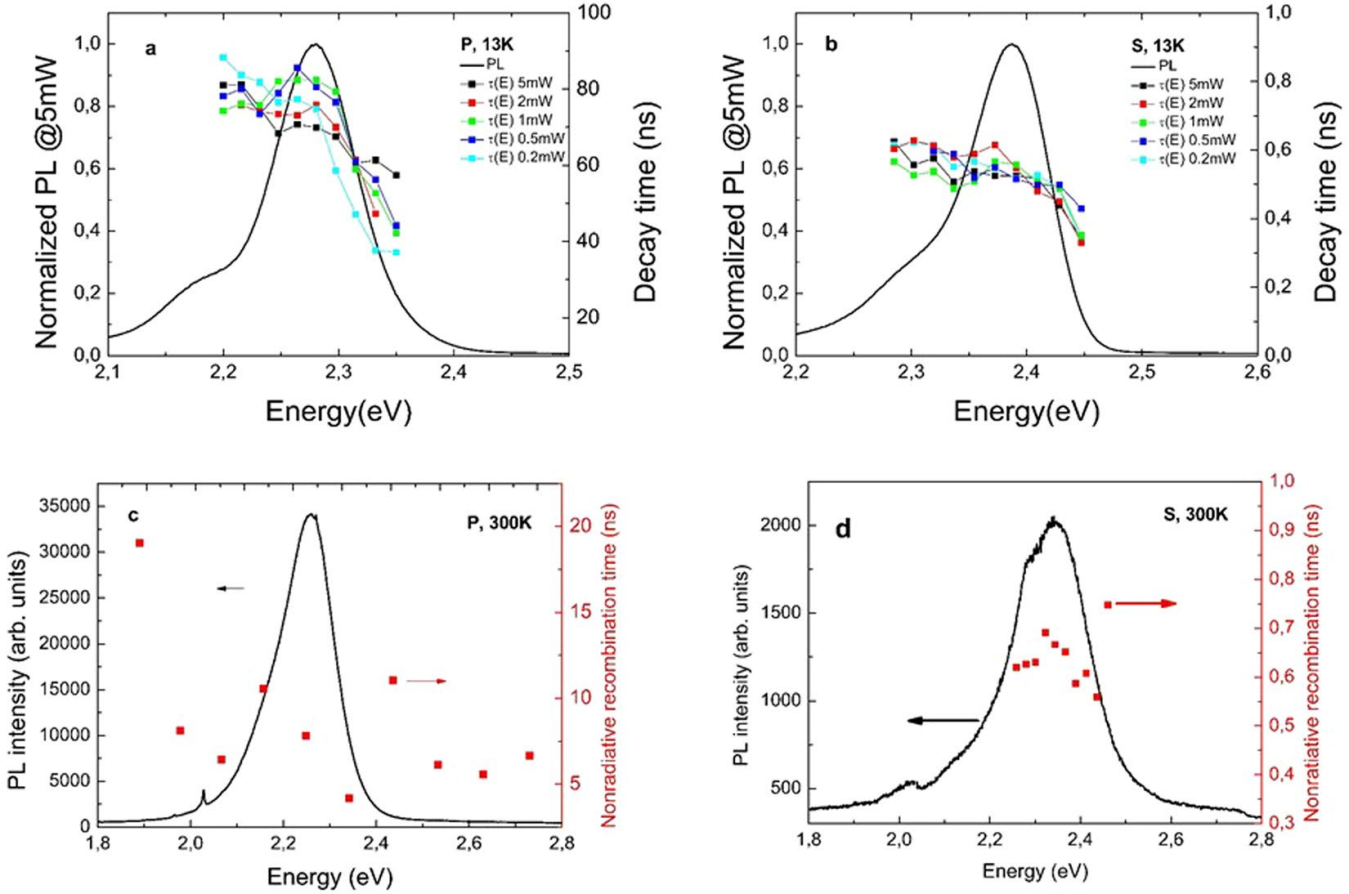
Dispersion of radiative recombination time at (**a**) LT in *P* sample, (**b**) LT in *S* sample, (**c**) RT in *P* sample, (**d**) RT in *S* sample.

In Fig. 7a–d we show the dispersion of the PL decay times at low (LT) and room temperature (RT), for polar (*P*) and semipolar (*S*) structures. Please note that, generally, at LT the recombination has almost uniquely radiative component. Looking at Fig. 2a,b, we observe a pronounced difference between the samples: in the case of *P* sample the recombination lifetime decreases steadily with the recombination energy while for *S* sample we observe much weaker dispersion of the recombination time. It would be natural to associate increase of carrier lifetime with decreasing energy with the presence of electric field, which slows down the recombination and red shifts emission energy. At RT the recombination is dominated by the nonradiative component. We observe, that at RT (Fig. 2c,d) the recombination time is almost independent of the emission energy for both *P* and *S* structure. This means, that inhomogeneity existing in the crystal does not influence the process of nonradiative recombination due to Shockley-Hall-Read recombination.

**Figure 8:**
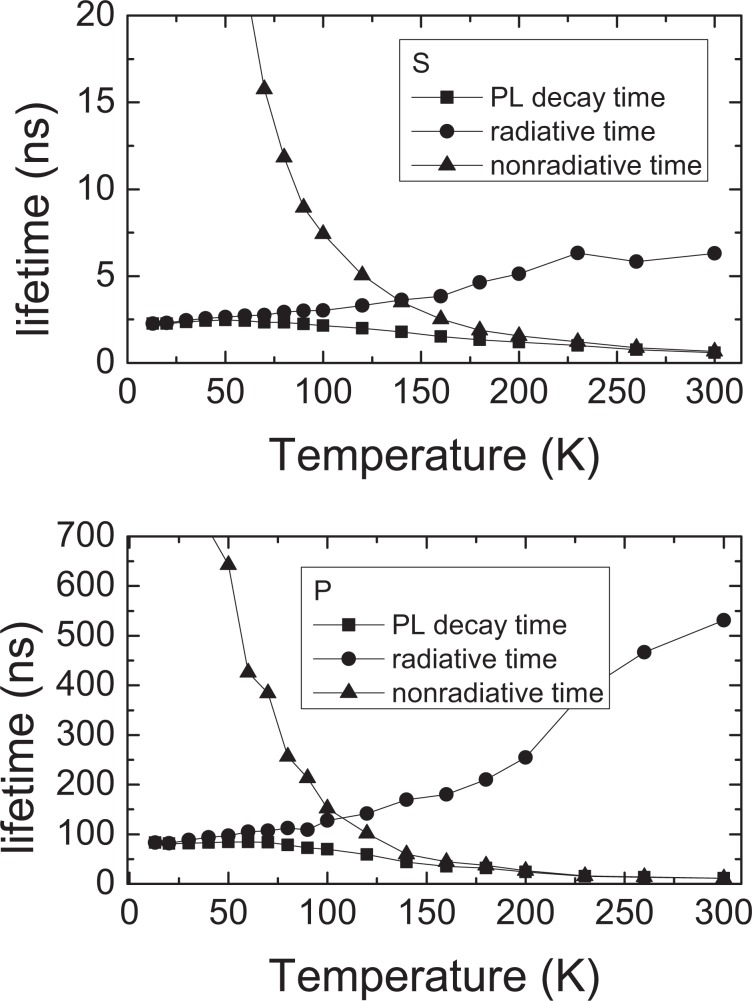
Radiative and nonradiative recombination time vs. temperature in InGaN QW structures, (**a**) sample *P*, (**b**) sample *S*.

Is usefull to calculate separetely radiative and nonradiative recombination time. We can do it by measuring the temperature dependence of the recombination time and using the expressions:

4 $${\rm{\eta }}=\frac{{{\rm{\tau }}}_{{\rm{PL}}}}{{{\rm{\tau }}}_{{\rm{rad}}}}$$

5 $${\rm{\eta }}=\frac{{{\rm{I}}}_{{\rm{LT}}}}{{{\rm{I}}}_{{\rm{T}}}}$$

we can plot both, radiative and nonradiative recombination time versus temperature (see Fig. 8).

It is worth to notice that both, radiative and nonradiative recombination times for P and S samples differ by almost two orders of magnitude. The fact that nonradiative recombination is much slower in *P* is inconsistent with Langer’s model, which assumes independence of SHR recombination from internal electric field.

The nonradiative recombination starts to dominate over the radiative component at about 100 K for *P* sample and about 150 K for *S* sample.

## Discussion

To get a new insight into radiative and nonradiative recombination mechanizms we tried to select out two phenomena: the internal electric field, and composition fluctuations effect. To this aim we compared PL dynamics for InGaN laser structures grown on two different crystallographic planes of GaN substrate: polar c-plane (*P* samples) and semipolar (20–21) plane (*S*-samples).

We can conclude the obtained results by the following statements:

1. Measured FWHM by CL over large range of analyzed area, for both type of samples (P,S), revealed almost no change. A possible explanation of an emission line broadening could be related to submicron size inhomogeneities which are besides of the cathodoluminescence analysys resolution.
2. The lifetime of the radiative and nonradiative recombination is much longer in the polar InGaN QW structures comparing to the semipolar ones. This is an obvious result in the case of radiative recombination since QCSE leads to decreased transition probability. However, it is less obvious result for SHR nonradiative recombination, as it is not clear how this mechanism is susceptible to the electric fields. We observed that SHR effect is energy independent, meaning that SHR has the same recombination time in all regions and for all mechanism providing emission of light. This is similar to the way of arguing used by Langer *et al*.^17^, who assumed that SHR recombination is independent from local electric field, contrary to the Kioupakis *et al*.^28^ predictions of strong dependence of the SHR recombination on electric field.
3. Measured PL decay times indicate that at low temperature in polar samples, excitonic recombination governs light emission, however, suprisingly, in semipolar case bimolecular recombination is the most visible. Our explanation of this phenomenon assumes that in semipolar QWs excitons are almost free and not so stable as in the case of polar QWs. We can predict that in this case excitonic recombination would not be so effective like bimolecular one.
4. Observed strong energy dispersion of the radiative lifetimes for polar sample and very weak dispersion for the semipolar one seems to support the Langer model, that links this dispersion with regions of QWs characterized by different levels of QCSE. However, this is inconsistent with the fact that nonradiative recombination seems to be much slower in polar structure and thus dependent on the amount of the electric field. The above observation suggests that the despersion depends more on carrier localization than on internal electric field.

## Methods

### Samples

To perform experimental analysis by various characterization techniques we have used polar (*P*) and semipolar (*S*) sets of samples. *P* samples were grown on conventional *c-*plane GaN and *S* samples on (20–21) plane GaN. Ammono-GaN substrates of low dislocation density (10^4^ cm^−2^)^29^ were used. The samples were fabricated in the same growth run by Metalorganic Vapor Phase Epitaxy (MOVPE). Epitaxial structure consists four In_0.22_Ga_0,78_N, 2.8 nm thick QWs and four 6.8 nm thick quantum barriers (QBs). The active region is sandwiched between two In_0,04_Ga_0,96_N waveguides layers, the first one (100 nm) of *p*-type, doped with Si and the other one (60 nm) of n-type, doped with Mg. On the top of the upper waveguide layer 20 nm thick Al_0.15_GaN:Mg, acting as an electron blocking, layer was placed. The structure was completed with 300 nm p-type GaN:Mg. These structures emitted green light (530–550 nm).

### Experimental techniques

In PL measurements samples were excited by 400 nm frequency-doubled pulses (150 fs at 4.25 MHz) coming from a Ti:Sapphire laser. The average power of exciting beam was 1 mW. The PL signal was dispersed by a 0.3 m focal length spectrometer equipped with 300 gr/mm diffraction grating and detected using nitrogen cooled silicon CCD array.

CL was measured using using Hitachi SU-70 scanning electron microscope equipped with Horiba Jobin Yvon optical detection system.

## Data Availability

The datasets generated and analysed during the current study are available from the corresponding author on reasonable request.
